# Microbial disruption in the gut promotes cerebral endothelial dysfunction

**DOI:** 10.14814/phy2.15100

**Published:** 2021-11-09

**Authors:** April J. Rustia, James S. Paterson, Giles Best, Elke M. Sokoya

**Affiliations:** ^1^ Chronic Disease Research Laboratory Flinders Health and Medical Institute College of Medicine and Public Health Flinders University Bedford Park South Australia Australia; ^2^ Microbial Systems Laboratory College of Science and Engineering Flinders University Bedford Park South Australia Australia; ^3^ Flow Cytometry Facility Flinders Health and Medical Research Institute College of Medicine and Public Health Flinders University Bedford Park South Australia Australia

**Keywords:** antibiotics, cerebral, endothelium, microbiota, nitric oxide

## Abstract

Cerebrovascular disease is a group of conditions characterized by disorders of the cerebral vessels. Endothelial dysfunction renders the vasculature at risk of impaired blood flow and increases the potential of developing cerebrovascular disease. The gut microbiota has been recently identified as a possible risk factor of cerebrovascular disease. However, a direct link between gut microbiota and cerebral vascular function has not been established. Therefore, the aim of this study was to determine the effect of gut bacterial disruption on cerebral endothelial function. Male inbred Sprague‐Dawley rats were randomly assigned to receive either drinking water with (*n* = 4) or without (*n* = 4) a cocktail of nonabsorbable broad‐spectrum antibiotics (streptomycin, neomycin, bacitracin, and polymyxin B). Three weeks of antibiotic treatment resulted in a significant reduction in bacterial load and shifts within the bacterial sub‐populations as assessed using flow cytometry. Using pressure myography, we found that spontaneous tone significantly increased and L‐NAME‐induced vasoconstriction was significantly blunted in middle cerebral arteries (MCAs) harvested from antibiotic‐treated rats. ATP‐mediated dilations were significantly blunted in MCAs from antibiotic‐treated rats compared to their control counterparts. Immunoblotting revealed that the eNOS‐P/total eNOS ratio was significantly reduced in cerebral artery lysates from antibiotic‐treated rats compared to controls. Our findings suggest that disruption of the gut microbiota leads to cerebral endothelial dysfunction through reduction of eNOS activity. This study highlights the potential of the microbiota as a target to reverse endothelial dysfunction and a preventative approach to reducing risk of stroke and aneurysms.

## Graphical abstract

Our study provides direct evidence suggesting that gut dysbiosis can lead to endothelial dysfunction in the resistance‐size vessels of the brain. It highlights the gut microbiota as a preventative strategy to mitigate the risk of stroke.

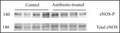

## 1 INTRODUCTION

Cerebrovascular disease is the fifth leading cause of premature death in the US (Centers for Disease Control & Prevention, National Center for Health Statistics, 2020). It refers to a group of conditions, such as stroke and aneurysms that affect the blood vessels and blood supply to the brain. The inner lining of blood vessels is comprised of a monolayer of endothelial cells. Not only do they act as a physical barrier between the blood and the tissue, but also endothelial cells influence blood vessel diameter changes by secreting various agents such as nitric oxide (NO). Damage to the endothelial cells, called endothelial dysfunction, is a strong predictor of future clinical events such as stroke and Alzheimer's disease (Schachinger et al., 2000). Therefore, understanding how endothelial dysfunction occurs, will allow us to develop strategies to prevent, or at least reduce, the mortality associated with these clinical events.

Our gastrointestinal tract contains trillions of small organisms referred to as the “gut microbiota” (Clemente et al., 2012). Most of these organisms are bacteria and they assist with essential functions, including the breakdown of complex molecules in our food, the support and programming of our immune system, and the transmission of signals to the brain thereby influencing mood and memory (Sherwin et al., 2016). An unbalanced gut microbiota, or dysbiosis, has been linked to many diseases including asthma, obesity, cancer, and cardiovascular disease (Clemente et al., 2012). In animal studies, microbiota‐depletion with broad‐spectrum antibiotics led to significantly increased stroke‐induced mortality (Winek et al., 2016) and restoring the balance of gut microbes improved stroke outcome (Singh et al., 2016).

The activity of gut bacteria leads to the release of various agents that can potentially affect the function of endothelial cells in peripheral conduit blood vessels (Tang et al., 2017). First, short‐chain fatty acids (SCFAs), including butyrate, acetate, and priopionate, are produced from the fermentation of indigestible dietary fibers (Bedford & Gong, 2018). SCFAs can cross the intestinal epithelial barrier and enter the circulation. Here they bind to G protein‐coupled free fatty acid receptors on the endothelium, thereby inhibiting nuclear factor‐kappa B (NF‐kB), a transcription factor which drives the production of pro‐inflammatory cytokines (Li et al., 2018; Vinolo et al., 2011). Second, the metabolism of aromatic amino acids, such as tryptophan, phenylalanine, and tyrosine, in the gut, produces uremic toxins such as indoxyl sulfate, phenyl sulfate, and hippuric acid. These toxins generate reactive oxygen species and promote endothelial dysfunction (Amedei & Morbidelli, 2019). Third, the metabolism of dietary choline, L‐carnitine, and betaine, produces trimethylamine that is, oxidized in the liver to form trimethyl‐N‐oxide (TMAO). TMAO promotes vascular inflammation through activation of endothelial and smooth muscle cell MAPK and NF‐kB signalling (Seldin et al., 2016). Finally, the gut microbiota produce a number of gases, including NO and hydrogen sulfide (H_2_S). NO produces either vasodilation or vasoconstriction, depending upon its in vivo concentration. At nanomolar concentrations, eNOS‐derived NO facilitates basal tone and resting blood flow. At micromolar concentrations, however, inducible NOS (iNOS)‐derived NO is cytotoxic. Increased permeability of the intestinal epithelial barrier allows the movement of lipopolysaccharide (LPS), found in the wall of Gram‐negative bacteria, from the gut lumen into the circulation. LPS activates iNOS to produce large amounts of NO which then reacts with oxygen radicals to produce peroxynitrite, leading to vasoconstriction (Münzel et al., 1997). H_2_S produces vasodilation through upregulating eNOS and opening ATP‐sensitive potassium channels (Bełtowski & Jamroz‐Wiśniewska, 2014).

Endothelium‐derived NO is critical to the normal functioning of the endothelium (Förstermann & Münzel, 2006) and a defining feature of cerebrovascular disease is reduced endothelium‐derived NO (Zhu et al.,^2016^). Work in peripheral blood vessels, suggests a role of gut bacteria in mediating endothelial function (Karbach et al., 2016; Vikram et al., 2016). However, no studies to date have characterized the effects of the gut microbiota on endothelial function in the brain. Therefore, the aim of this project was to identify the role of the colonic bacterial community in contributing to the regulation of endothelial function in cerebral arteries.

## 2 MATERIALS AND METHODS

### 2.1 Animal model of dysbiosis

All procedures were approved by the Flinders University Animal Ethics Committee (approval number 947‐17). Male inbred Sprague‐Dawley rats were bred in the Flinders Medical Centre Animal Facility. Eight rats were weaned at 3 weeks of age and randomly assigned into control (*n* = 4) and antibiotic‐treated groups (*n* = 4). In order to homogenize their gut flora, rats within each experimental group were co‐housed in autoclaved individually ventilated cages. Enrichments were absent from the cages of all rats and their parents used in the study to prevent gut exposure to external environmental factors. Animals had ad libitum access to autoclaved standard rat chow and drinking water throughout the study.

**FIGURE 1:**
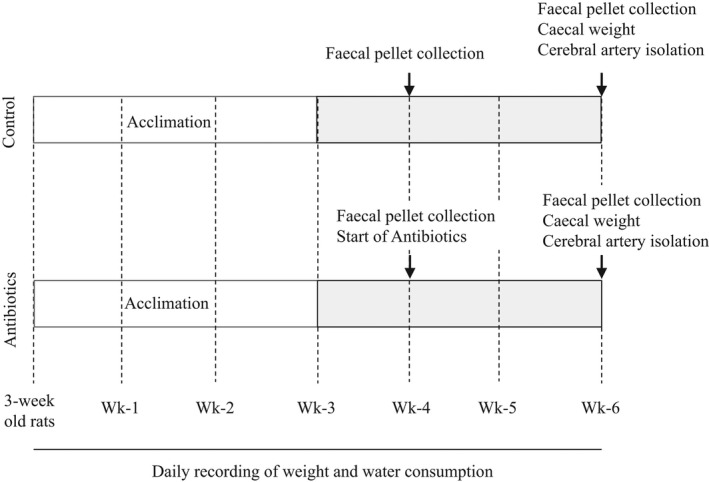
Schematic figure illustrating the timeline of the experimental protocol

After 3 weeks of acclimation, animals received autoclaved tap water with (antibiotic‐treated; *n* = 4) or without (control; *n* = 4) an antibiotic cocktail (see Table 1) for 3 weeks. A cocktail of nonabsorbable antimicrobials were used to reduce the total bacterial load and alter the bacterial community distribution pattern. The antibiotic cocktail contained streptomycin (120 mg/kg/day), neomycin (60 mg/kg/day), polymyxin B (60 mg/kg/day), and bacitracin (120 mg/kg/day). The choice of antibiotics was based on the study by (Lam et al., 2016) who showed that administration of streptomycin, neomycin, bacitracin, and polymyxin B significantly reduced total microbial numbers in rats. These agents have been previously shown to be either not absorbed or poorly absorbed from the intestine (Gupta et al., 2009; Stebbins et al., 1945; Waaij et al., 1974; Waisbren & Spink, 1950). The water bottles were covered with foil to protect the activity of the antibiotics that were light‐sensitive. Animal weight and water consumption were recorded daily. Animals were also monitored for movement, pain (facial grimace), evidence of dehydration, diarrhea, and well‐being (ruffling and hunching). The experimental timeline is provided in Figure 1.

**TABLE 1:** Characteristics of antibiotics

Antibiotic	Dose [12]	Class	Bacterial targets
Streptomycin	120 mg/kg/day	Aminoglycoside	Broad spectrum, Gram‐negatives
Neomycin	60 mg/kg/day	Aminoglycoside	Broad spectrum, Gram‐negatives
Bacitracin	120 mg/kg/day	Polypeptide	Narrow spectrum, Gram‐positives
Polymyxin B	60 mg/kg/day	Cationic, cyclic polypeptide	Narrow spectrum, Gram‐negatives

### 2.2 Preparation of antibiotics

Stock solutions of streptomycin (20 mg/ml), neomycin (50 mg/ml), and polymyxin B (50 mg/ml) were prepared by dissolving each antibiotic powder in filter sterilized Milli‐Q water. Aliquots were frozen and stored at −20°C in the dark. Bacitracin was prepared fresh daily due to its lack of stability in solution. The antibiotic‐treated water was changed daily to maintain optimal antibiotic activity.

### 2.3 Fecal sample preparation for flow cytometry

Flow cytometry was used to analyze the bacterial load and sub‐population structure within rat fecal pellet samples. The method was adapted from previously published work (Koch et al., 2013; Vandeputte et al., 2017; Waaij et al., 1994). For staining of bacterial cells, a 0.2% v/v working stock solution of SYBR Green (ThermoFisher, S7563) was prepared with Milli‐Q water and aliquots were stored at −20°C in the dark. A 0.028% v/v bead stock solution (ThermoFisher, F8823) was prepared using Milli‐Q water and stored at 4°C in the dark. A 0.1% v/v bead stock working solution was made from diluted bead stock solution and filter‐sterilized Milli‐Q water and stored at 4°C in the dark.

Fresh fecal pellets were obtained from each rat at the beginning of the study and then 3 weeks later following water consumption with or without antibiotics. After weighing the pellet, it was immediately added to sterile PBS (1 ml per 10 mg of fresh fecal weight). The pellet was homogenized using an IKA Ultra Turrax‐T10 homogenizer for 5 min, filtered through a 40 μm cell strainer and centrifuged at 3008 g for 10 min at 4°C. The pellet was washed three times with 20 ml ice‐cold filter‐sterilized PBS. After the third wash, the supernatant was discarded and the pellet was resuspended in 980 μl of ice‐cold sterile PBS and 20 μl of 25% glutaraldehyde fixative (0.5% final concentration) was added. The fecal bacteria samples were left to fix for 30 min on ice in the dark. The fixed samples were vigorously vortexed and a small amount from each fixed sample was added to 3 ml PBS. The amount of fixed sample that was added to PBS was dependent on the wet weight of the fecal pellet. 50 or 100 μl of fixed sample was added to 3 ml PBS when the wet fecal pellet weight was <25 or ≥25 mg, respectively. The fixed sample was washed three times with 3 ml ice‐cold sterile PBS and resuspended in 3 ml ice‐cold sterile PBS. Serial dilutions were performed to produce 1:10, 1:100, and 1:1000 samples. Two unstained (300 μl sample) and two stained samples (500 μl sample +12.5 μl 0.2% v/v SYBR Green Working Stock Solution) were made from each of these three diluted samples. Two microfuge tubes containing 500 μl sterile PBS and two microfuge tubes containing 500 μl sterile PBS +12.5 μl 0.2% v/v SYBR Green Working Stock solution were prepared as “noise tubes”. All tubes were then incubated in the dark at room temperature for 10 min. Events were recorded for each sample in triplicate.

### 2.4 Detecting bacteria in fecal samples using flow cytometry

Bacteria present within the fecal samples were identified using a flow cytometer (CytoFLEX S, Beckman Coulter) and small particle detector settings. Optimal detection of bacteria based on size and fluorescence were established using the violet side scatter (VSSC) and SYBR green fluorescence, respectively. The SYBR Green stain was excited at a wavelength of 488 nm and produced fluorescence emission at 520 nm. Within the cytograms generated using the CytExpert version 2.0 software (Beckman Coulter), gates were applied to separate bacteria from background, small non‐bacterial particles, and unstained bacteria based on particles detected in PBS and unstained controls. A total of 20 s of data were recorded from each sample. The instrument and gating settings were consistent for the measurement of all samples.

### 2.5 Analysis for total bacterial load

Total bacterial load was determined by calculating the number of SYBR green positive events in stained cells/μl and subtracting the mean of the number of positive events in unstained cells/μl. The number of events/μl in the fixed sample was calculated according to the dilution factor and data were normalized to the wet weight of the fecal pellet. A log_10_ transformation was then performed prior to statistical analysis.

### 2.6 Analysis for bacterial sub‐populations

Five distinct bacterial sub‐populations were identified based on their light scatter characteristics and staining with SYBR green. Changes in the number of bacteria in each of the five populations over the 3‐week period were assessed in samples from the control and antibody‐treated rats. The number of events within each of these smaller gates was used to calculate the number of events/μl in the fixed sample as described earlier according to the dilution factor and normalized to the wet weight of the fecal pellet. A log_10_ transformation was then performed prior to statistical analysis.

### 2.7 Collection of trunk blood

At the time of harvesting the cerebral vessels, the rat was placed in an anesthetic chamber containing 5% isoflurance and allowed to spontaneously breathe. Deep anesthesia was confirmed using the hind limb and tail pinch test. The rat was euthanized by decapitation using a rodent guillotine. Trunk blood was collected into heparinized centrifuge tubes, immediately inverted to mix the blood and centrifuged at 2000 g. The plasma was carefully aspirated and stored in 100 μl aliquots at −20°C. Total cholesterol, high‐density lipoprotein (HDL), and C‐reactive protein (CRP) were measured using a Cobas 8000 modular analyzer (Roche).

### 2.8 Harvesting cerebral blood vessels and cecum

Following decapitation and trunk blood collection, the brain was carefully removed and placed in a dish containing ice‐cold physiological saline solution (PSS). The right and left middle cerebral arteries (MCAs) were harvested and cleaned of surrounding connective tissue. The remaining cerebral arteries including the basilar artery, anterior, and posterior communicating arteries were harvested, pooled and placed in 140 μl ice‐cold protein extraction buffer containing 50 mM Tris, pH 7.8, 150 mM NaCl, 1% Triton‐X, 0.5% NP‐40, 0.25% DOC containing 1 mM PMSF, and a protease inhibitor tablet with EDTA (Roche). The pooled arteries were then homogenized, centrifuged, aliquoted, and stored at −80°C. Finally, the cecum was carefully removed, photographed, and weighed.

### 2.9 Pressure myography

A series of in vitro functional tests using pressure myography were performed on MCAs to determine the effect of antibiotic‐induced colonic dysbiosis on endothelial function. In separate experiments, the right and left MCA from each rat was cannulated from each end with micropipettes in a custom‐built pressure myograph chamber. PSS was perfused luminally and circulated abluminally and the temperature maintained at 37°C. The mounted vessel was tested for leaks and vessels that were unable to hold pressure were discarded. Intraluminal pressure was set at 85 mm Hg with a flow rate of 150 μl/min and the vessel was allowed to equilibrate and develop spontaneous tone for 1 h. During this equilibration period, the vessel constricted from its fully dilated diameter at initial pressurization. The vessel chamber was positioned on the stage of an inverted microscope equipped with a video camera to allow real time viewing of the mounted vessels. Concentration response curves to luminal ATP (endothelium‐dependent dilations) and abluminal MAHMA NONOate (smooth muscle‐dependent dilations) were recorded from the two MCAs, respectively.

### 2.10 Pressure myography reagents

L‐NAME, ATP, MAHMA NONOate, and PSS components were purchased from Sigma and 2MeSATP was purchased from Tocris. Stock solutions of MAHMA NONOate were made in 0.01 M NaOH and the remaining stock solutions were made in water. PSS contained (in mM) 119 NaCl, 4.7 KCl, 22.8 NaHCO_3_, 1.2 KH_2_PO_4_, 1.2 MgSO_4_, 0.026 EDTA, 1.6 CaCl_2_, and 5.5 glucose. Calcium‐free PSS contained (in mM) 119 NaCl, 4.7 KCl, 22.8 NaHCO_3_, 1.2 KH_2_PO_4_, 1.2 MgSO_4_, 0.026 EDTA, 5.5 glucose, and 1.0 EGTA.

### 2.11 Assessment of endothelial function

After the development of spontaneous tone, basal production of NO was determined by recording the constriction in response to luminal and abluminal L‐NAME (3 × 10^−5^ M) exposure. These MCAs were then assessed for smooth muscle function (see Assessment of Smooth Muscle Function). In another set of MCAs, agonist‐stimulated endothelial function was assessed by performing concentration response curves to luminal exposure of ATP (10^−7^ to 10^−4^ M). At the end of each experiment, the arteries were exposed to calcium‐free PSS containing 1 mM EGTA in order to record the maximum dilation of the vessel.

### 2.12 Assessment of smooth muscle function

Vascular smooth muscle cell function was examined by recording MCA diameter changes in response to abluminal NO exposure. Following luminal and abluminal incubation with L‐NAME (3 × 10^−5^ M), concentration response curves to abluminal (Z)‐1‐[N‐methyl‐N‐[6‐(N‐methylammoniohexyl)amino]]diazen‐1‐ium‐1,2‐diolate (MAHMA NONOate; 10^−9^ to 10^−4^ M) were recorded.

### 2.13 Reporting of vasodilator responses

To account for the variations in artery size between animals, the vasodilator responses were expressed as a percentage of the maximum diameter of each vessel in the presence of calcium‐free PSS. This was calculated using the following formula:

$$\text{Dilation}\left(\%\right)=\frac{\left(\text{Final diameter}‐\text{Initial diameter}\right)}{\left(\text{Maximum diameter}‐\text{Initial diameter}\right)}\times 100.$$

The final diameter was recorded as the steady state diameter that was reached after exposure to each concentration of ATP and MAHMA NONOate. The initial diameter was the baseline diameter of the vessel before the exposure to each concentration of ATP and MAHMA NONOate. The maximum diameter was the diameter recorded in the pressure of calcium‐free PSS containing 1 mM EGTA.

### 2.14 Immunoblotting

Immunoblotting was performed in lysates from pooled cerebral arteries (basilar artery, anterior, and posterior communicating arteries). Protein expression of eNOS‐P and total eNOS was measured as previously described (Tajbakhsh et al., 2015). The primary antibodies were mouse anti‐eNOS monocloncal primary antibody (BD Transduction Labs, 610296 at 1:1000 dilution) or rabbit anti‐eNOS‐P polyclonal primary antibody (Cell Signaling Technology, 9571 at 1:400 dilution). In pilot studies, the antibody specificity was established by confirming a band at the appropriate molecular weight in positive controls (HUVEC and eNOS‐transfected human embryonic kidney (HEK) cells) and the absence of the band in negative controls (HEK cells). Total protein loaded on the gel was transferred to a low fluorescence PVDF membrane and captured using a BioRad EZ‐Doc Imager and analyzed using Image Lab 4.0.1 software (Bio‐Rad Laboratories). This method of protein normalization has previously been shown to be superior to total protein stains such as Coomassie Blue or SYPRO Ruby, or antibody loading controls (Colella et al., 2012). Immunoreactive bands were detected by enhanced chemiluminescence, captured using a LAS‐4000 imager (Fujifilm) and quantified using Image Quant LAS‐4000 (Carestream Molecular Imaging software). Immunoreactive bands to eNOS and eNOS‐P were expressed relative to total protein.

### 2.15 Statistical analysis

All data are presented as mean ± standard error of the mean (SEM). Statistical analysis was performed using paired (total bacterial load and bacterial sub‐populations) and unpaired *t*‐tests (cecal weights and densitometry) and two‐way repeated measures ANOVA with post hoc Bonferroni tests (rat weight, water consumption, and pressure myography concentration response curves) using GraphPad Prism v5.04 (GraphPad software). Results were considered significant when *p *< 0.05.

## 3 RESULTS

### 3.1 Effect of antibiotic treatment on animal phenotype

**FIGURE 2:**
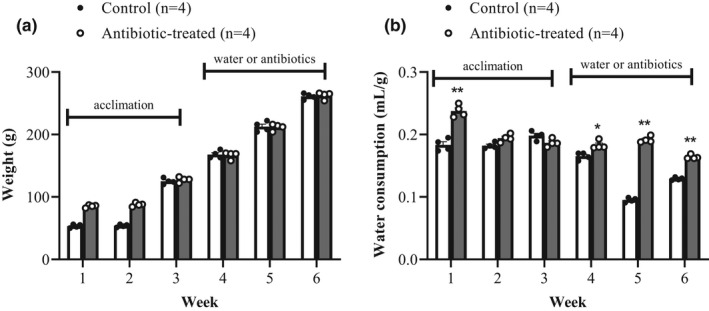
Weight gain over time in control and antibiotic‐treated rats (a). Weight gain, expressed as the mid‐week weight, over the entire experimental period of control (*n* = 4) and antibiotic‐treated (*n* = 4) male rats. Data are expressed as mean ± standard error of the mean (SEM). The first three weeks represent the homogenization period and the antibiotic treatment commenced in week 4. Weight gain was analyzed using a two‐way RM ANOVA using GraphPad Prism v5.04. Water consumption over time in control and antibiotic‐treated rats (b). Mid‐week water consumption, normalized to body weight (ml/g body weight) of control (*n* = 4) and antibiotic‐treated (*n* = 4) male rats. *indicates *p* < 0.01 and **indicates *p *< 0.0001 versus control. Data were analyzed using 2‐way RM ANOVA with GraphPad Prism v5.04

To alter the intestinal microbiota, rats were subjected to a cocktail of broad‐spectrum nonabsorbable antibiotics in their drinking water for 3 weeks. Antibiotic treatment did not adversely affect the well‐being of the rats as assessed by ruffling and hunching. There was no evidence of pain as assessed by facial grimace. Weight gain was comparable between control and antibiotic‐treated rats (Figure 2a). At the end of the study, animals weighed 261 ± 2.3 g and 262 ± 2.8 g in control and antibiotic‐treated rats, respectively.

**FIGURE 3:**
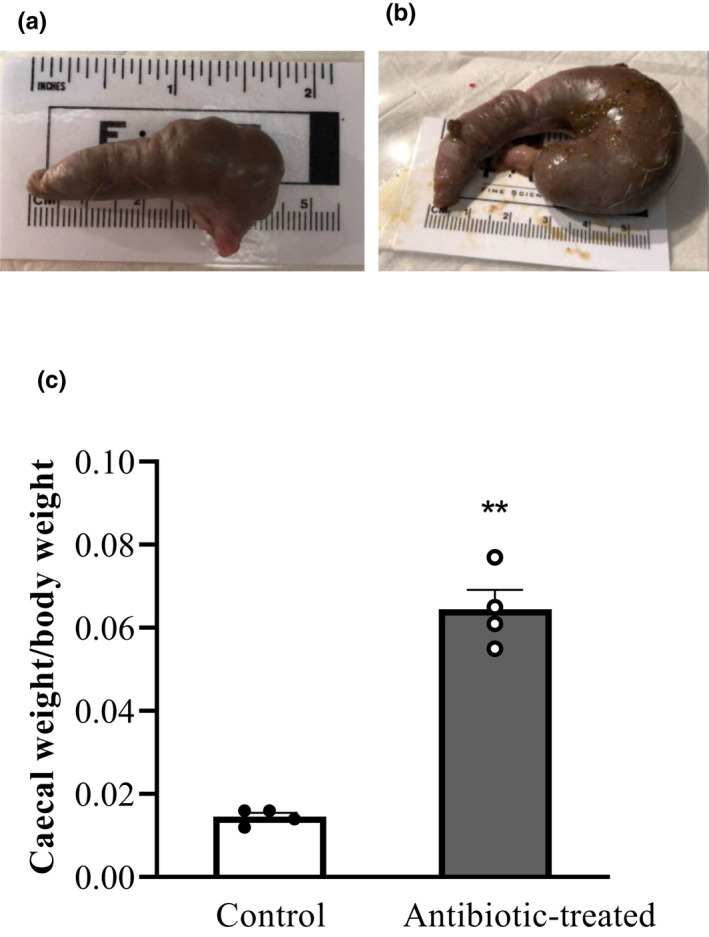
Caecal weight to body weight ratio of control and antibiotic‐treated rats. Representative photographs of the caecum harvested from control (a) and antibiotic‐treated (b) male rats. The average caecal weight of control and antibiotic‐treated rats normalised to body weight expressed as mean ± SEM (c). The difference was analyzed using a two‐tailed unpaired t‐test with 95% confidence interval with GraphPad Prism. **indicates *p *< 0.0001 versus control

Water consumption significantly increased in the antibiotic‐treated cohort in week 1 of the homogenization period (*p *< 0.0001) and in weeks 4, 5, and 6 after antibiotics were added to the drinking water (*p *< 0.01, *p* < 0.0001, *p* < 0.0001, respectively; Figure 2b). Furthermore, at the end of the study, cecal weight (normalized to body weight) was significantly larger in antibiotic‐treated rats (0.065 ± 0.005) compared to controls (0.014 ± 0.001; *p *< 0.05) (Figure 3c). This represents a 3.5‐fold increase in cecal weight after antibiotic treatment. The differences in gross morphology are clearly visibly in the photographs (Figure 3a,b). In antibiotic‐treated rats, the cecum is visibly longer and enlarged compared to control rats.

### 3.2 Effect of antibiotic treatment on bacterial load

**FIGURE 4:**
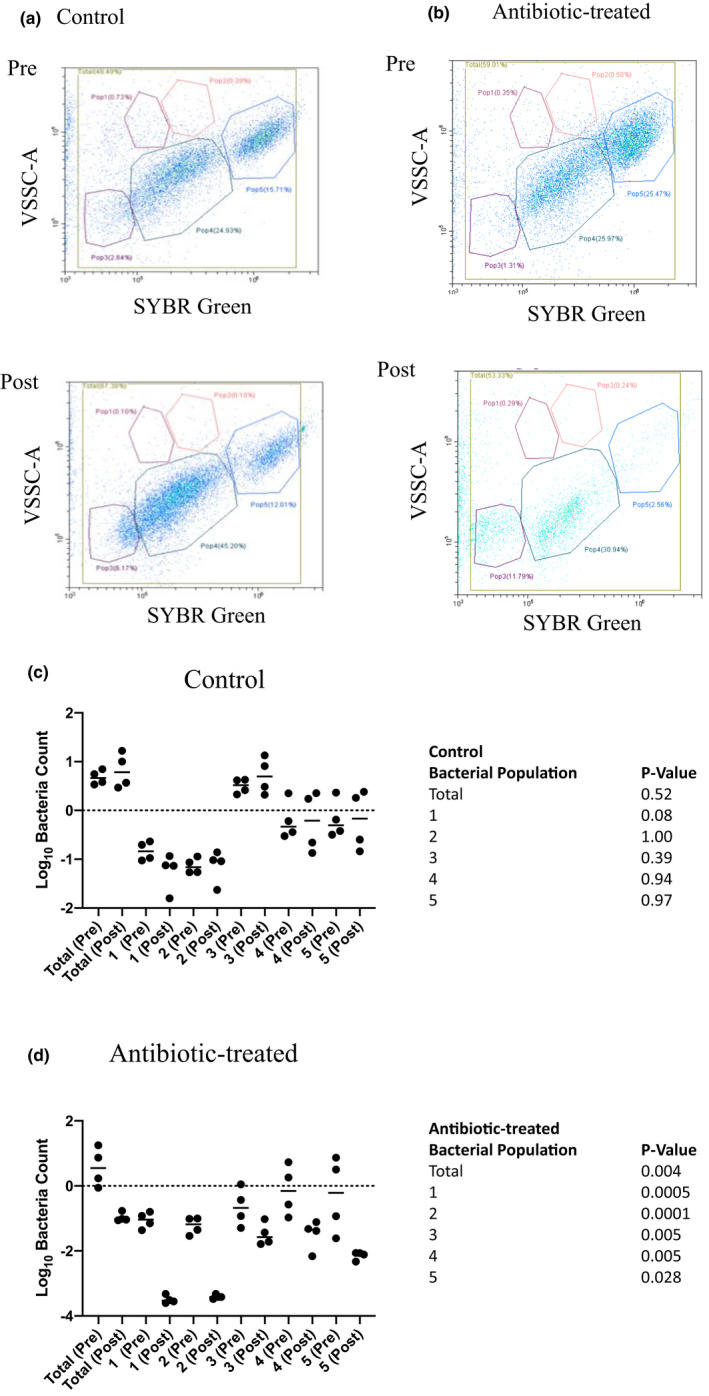
Total bacterial load and sub‐population structure in control and antibiotic‐treated male rats. Representative cytograms of bacteria in faecal pellets obtained prior to and three weeks following control (a) or antibiotic treatment (b) are shown on the left. The five individual populations are delineated. The log_10_ transformation of the number of events in the fixed samples obtained from control (c) and antibiotic‐treated rats (d) is shown in the histograms on the right. Statistical analysis was performed using a paired t‐test and *p*‐values comparing the total bacterial load and bacteria number in each of the five populations, pre and post control or antibiotic‐treatment period, are indicated.

The total bacterial load was determined in fecal pellets prior to and following water consumption with or without antibiotics. Representative images showing the gates applied to enumerate the total bacterial load and bacteria in the five distinct populations is shown in Figure 4a,b. Figure 4c,d show the data from all animals in the control and antibiotic‐treated groups. A significant (*p* = 0.004) decrease in total bacterial load was observed in antibiotic‐treated animals but not in control animals (*p *= 0.52).

### 3.3 Effect of antibiotic treatment on bacterial sub‐population structure

Five distinct bacterial sub‐populations were observed in fecal pellets from the control and pre‐antibiotic rats (Figure 4a,b). No significant changes in the bacterial count in any of the five sub‐populations were observed in the control animals over the 3 week period (Figure 4c). However, in the antibiotic‐treated animals, a consistent and significant decrease in the number of bacteria in each of the five sub‐populations was observed (Figure 4d). These data suggest that antibiotic treatment resulted in a significant reduction in the total bacterial load and a significant change in the bacterial sub‐population diversity.

### 3.4 Effect of antibiotic treatment on cerebral vascular function

As shown in Table 2, MCAs harvested from control and antibiotic‐treated animals had comparable maximum diameters. Spontaneous tone was significantly greater in antibiotic‐treated MCAs (35 ± 3%) compared to their control counterparts (30 ± 2%). In addition, L‐NAME‐mediated constriction was significantly blunted in antibiotic‐treated MCAs (10 ± 1%) compared to controls (17 ± 2%).

**TABLE 2:** Maximum diameter, percent tone, and L‐NAME‐mediated vasoconstriction in MCAs harvested from control and antibiotic‐treated rats

Experimental Group	Maximum diameter (μm)	Tone (%)	L‐NAME constriction (%)
Control (*n* = 4)	269 ± 6	30 ± 2.1	17 ± 1.6
Antibiotic‐treated rats(*n* = 4)	275 ± 5	35 ± 3.3*	10 ± 1.3*

Note

Values are reported as means ± SEM. Maximum diameter is the outer diameter recorded in the presence of calcium‐free PSS containing 1 mM EGTA.

* *p *< 0.05 compared to control.

**FIGURE 5:**
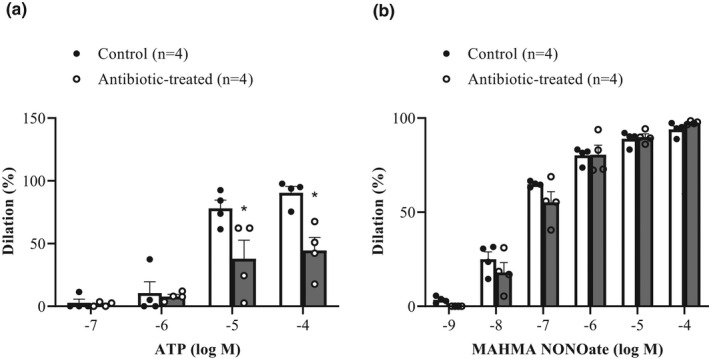
(a) Concentration response curves in MCAs harvested from control (*n* = 4) and antibiotic‐treated male rats (*n* = 4) to luminal application of ATP (10^–7^ to 10^–4^ M). The dilation to ATP was significantly blunted at the two highest concentrations of ATP in MCAs harvested from antibiotic‐treated rats compared to controls (*p *< 0.05; two‐way repeated measures ANOVA). (b) Concentration response curves in MCAs harvested from control (*n* = 4) and antibiotic‐treated rats (*n* = 4) to abluminal application of the NO donor, MAHMA NONOate (10^–9^ to 10^–4^ M) in the presence of 3 × 10^−5^ M L‐NAME. The dilation to MAHMA NONOate was comparable in MCAs harvested from control and antibiotic‐treated rats (*P* > 0.05)

Luminal administration of ATP (10^−7^ to 10^−4^ M) produced a concentration‐dependent dilation in isolated MCAs (Figure 5a). The dilation was significantly attenuated at the two highest concentrations of ATP in MCAs harvested from antibiotic‐treated rats compared to controls (*p *< 0.05; two‐way repeated measures ANOVA). In response to 10^−4^ M luminal ATP, MCAs dilated to 91 ± 5% in controls and 45 ± 10% in antibiotic‐treated rats.

The smooth muscle sensitivity to NO was compared in MCAs isolated from control and antibiotic‐treated rats by recording concentration‐dependent responses to the spontaneous NO donor, MAHMA NONOate (10^−9^ to 10^−4^ M) in the presence of L‐NAME (3 × 10^−5^ M) to eliminate endogenous sources of NO. As shown in Figure 5b, dilations to MAHMA NONOate were comparable in MCAs harvested from control and antibiotic‐treated rats.

### 3.5 Effect of antibiotic treatment on the phosphorylated eNOS to total eNOS ratio

**FIGURE 6:**
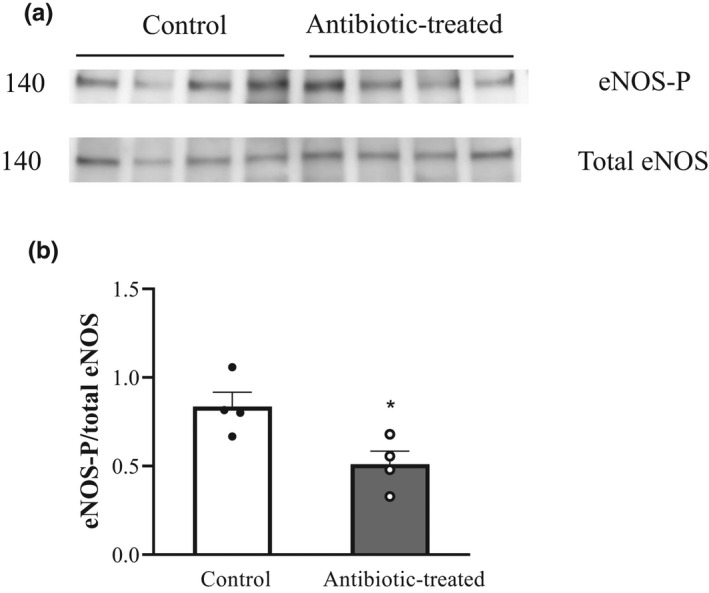
Representative immunoblots (a) showing the ratio of eNOS‐P protein expression and total eNOS protein expression in cerebral (pooled basilar artery, anterior and posterior communicating arteries) homogenates from control (*n* = 4) and antibiotic‐treated male rats (*n* = 4). A summary of immunoreactive band intensity is also shown (b). Values are expressed as intensity normalized to total protein (mean ± SEM). *p < 0.05; unpaired t‐test

The specificity of the primary antibodies was confirmed in lysates from HUVECs and eNOS‐transfected HEK cells (positive control) and HEK cells (negative control). Membranes probed separately with the antibody toward either eNOS‐P or total eNOS revealed a single band at the expected molecular weight of 140 kDa in the positive control (HUVECs and eNOS‐transfected HEK cells). No band was detected in the negative control (HEK cells). Total eNOS and eNOS‐P were detected in cerebral artery lysates obtained from control and antibiotic‐treated rats. Densitometric analysis revealed that the eNOS‐P/total eNOS ratio was significantly reduced in antibiotic‐treated rats (Figure 6b).

## 4 DISCUSSION

In the present study, we established an antibiotic‐treated rat model to induce colonic dysbiosis and assessed the effect on cerebral endothelial function. A 3‐week antibiotic regimen caused both a significant reduction in total bacterial load and a significant shift within the intestinal bacterial sub‐population structure, suggesting colonic dysbiosis. These changes within the microbiota were associated with reduced NO function within the MCA as evidenced by enhanced spontaneous tone, blunted L‐NAME‐mediated constriction, reduced luminal ATP‐mediated dilations, and reduced protein expression of the eNOS‐P to total eNOS ratio.

### 4.1 Effect of antibiotic treatment on phenotype

In order to reduce the intestinal bacterial load, a mixture of nonabsorbable antibiotics was added to the drinking water for 3 weeks. Supplementation of the drinking water with antibiotics did not adversely affect the health of the rats. Weight gain was comparable between control and antibiotic‐treated rats during the 6‐week experimental period (Figure 2a). Our findings align with previous studies in rats using the same antibiotic regimen (Lam et al., 2016) and a different antibiotic course of amoxicillin, cefotaxime, vancomycin, and metronidazole (Tulstrup et al., 2015). In contrast, another study demonstrated a lower body weight in C57Bl/6 mice treated for 6 weeks with antibiotics (Vikram et al., 2016). The different findings may be explained by the longer time course of antibiotic treatment (6 weeks vs. 3 weeks in our study) and/or the different antibiotic cocktail (metronidazole, ampicillin, neomycin, and vancomycin vs. streptomycin, neomycin, polymyxin B, and bacitracin in our study).

Daily water consumption was recorded to ensure that the antibiotic‐treated rats were receiving the correct antibiotic dosage. The first 3 weeks were an acclimation period in order to homogenize the gut microbes within each experimental group. Water consumption, normalized to body weight, was significantly greater in the pre‐antibiotic‐treated rats compared to controls in week 1 of the study (Figure 2b). However, in weeks 2 and 3, water consumption was comparable between the controls and pre‐antibiotic‐treated cohorts. Antibiotic treatment had not been initiated during this time and therefore, it is likely that the increased water consumption in week 1 was an anomaly. This may be a reflection of the environmental conditions and a move toward a heterogeneous microbiota within the cage. Antibiotic treatment resulted in significantly increased water consumption, normalized to body weight. Three weeks after the start of antibiotic treatment, rats consumed 0.0164 ± 0.002 ml/g body weight compared to 0.129 ± 0.001 ml/g body weight in controls. Our findings corroborate the results of Tulstrup and colleagues who showed that animals receiving different antibiotics to that used in the current study (either amoxicillin, cefotaxime, or metronidazole) had a higher water intake compared to their control counterparts (Tulstrup et al., 2015). In contrast, other studies have shown rodents consumed similar volumes of antibiotic‐treated and untreated water (Marx et al., 2014) and other studies have shown a temporary reduction in water consumption (Brunt et al., 2019; Reikvam et al., 2011), most likely due to the taste of the particular antibiotics used.

Antibiotic treatment resulted in a significantly increased cecum weight and size compared to controls (Figure 3). These findings are comparable to previous studies showing antibiotic‐mediated increases in cecum weight (Tulstrup et al., 2015; Zarrinpar et al., 2018). It also aligns with the larger cecum weight observed in germ‐free mice (Wostmann & Bruckner‐Kardoss, 1960). The cause of this enlargement of the cecum is unknown, although it has been speculated that it is mediated by the depletion of anaerobic bacteria (Skelly et al., 1962) through inhibiting water transport in the large intestine (Thompson & Trexler, 1971).

### 4.2 Effect of antibiotic treatment on bacterial sub‐population structure and load

Herein we have described a rat model of gut dysbiosis, whereby a 3‐week regimen of broad‐spectrum antibiotic consumption leads to a significant reduction in total bacterial load and a shift in the bacterial sub‐population structure. Fecal pellets were obtained prior to and following antibiotic treatment and used to assess total bacterial load and sub‐population structure. We found that a 3‐week exposure to a cocktail of antibiotics, resulted in a significant decrease in total bacterial load (Figure 4). The antibiotic cocktail also caused a remarkable shift in the gut microbial environment, as evidenced by the significant change in the number of bacteria in distinct sub‐populations observed by flow cytometry.

As a proxy for the evaluation of the gut microbiota, measuring the bacterial phenotype of fecal pellets has both advantages and limitations. The advantages are that samples are easily obtained and are non‐invasive, thereby allowing for multiple samples to be obtained. The limitations are that fecal pellets are a surrogate for the in vivo state. They comprise a mixture of commensal and transient bacteria from the entire gastrointestinal tract. Nevertheless, bacterial composition in fecal pellets provide a good approximation for bacterial load in the large intestine (Hillman et al., 2017).

While 16S rRNA gene sequencing can be used to profile bacterial communities (Greisen et al., 1994), it can be technically challenging and lacks the ability to discriminate between DNA from dead and live bacteria and between extracellular and intracellular DNA (Rogers et al., 2013; Wagner et al., 2008). To circumvent these limitations, the flow cytometry approach to bacterial enumeration has been validated in ecology (Koch et al., 2013), marine samples (Marie et al., 1997), human fecal samples (Vandeputte et al., 2017), and human sinuses (Cuesta‐Zuluaga et al., 2019). In addition, the workflow is faster, technically straightforward, cost effective, and very reproducible (Habtewold et al., 2016).

### 4.3 Effect of antibiotic treatment on cerebral endothelial function

Three weeks of antibiotic treatment resulted in endothelial dysfunction in the brain. First, spontaneous tone was significantly greater in antibiotic‐treated MCAs compared to their control counterparts (Table 3). Second, L‐NAME‐mediated constrictions were significantly blunted in antibiotic‐treated MCAs compared to controls (Table 3). The greater tone development and reduced constriction to L‐NAME could be either due to less basal NO being released from the endothelium as a result of lower basal eNOS activity or a decreased sensitivity of the smooth muscle to NO. It is more likely to be the former as antibiotic treatment did not affect MAHMA NONOate‐mediated dilations (Figure 5b) suggesting that smooth muscle sensitivity to NO is not compromised. Taken together, our results suggest that the basal pool of NO is significantly lower in antibiotic‐treated rats and this could be a reflection of decreased eNOS activity.

**TABLE 3:** Characterization of control and antibiotic‐treated rats. Data are expressed as mean ± SEM

Experimental Group	Blood glucose (mM)	Total cholesterol (mM)	HDL (mM)	Triglycerides (mM)	CRP (ng/ml)
Control (*n* = 4)	7.4 ± 0.38	3.4 ± 0.06	2.6 ± 0.07	1.8 ± 0.16	0.175 ± 0.050
Antibiotic‐treated rats (*n* = 4)	7.3 ± 0.32	3.4 ± 0.02	2.4 ± 0.02	1.4 ± 0.13	0.150 ± 0.019

Third, ATP‐mediated dilations were blunted in antibiotic‐treated MCAs compared to controls (Figure 5a). Previous studies from our laboratory have shown that the dilator response to ATP is abolished after removal of the endothelium and after preincubation with L‐NAME and indomethacin (Golding & Kepler, 2001) suggesting that the dilator response to ATP is mediated through both NO and EDH. L‐NAME alone, does not affect ATP‐mediated dilations at concentrations above 1 µM suggesting that the remaining dilations are elicited through endothelium‐dependent hyperpolarization (EDH) (You et al., 1997). This data therefore suggest that EDH is significantly blunted in MCAs isolated from antibiotic‐treated rats.

In addition to NOS‐derived NO, the enzymatic reduction of dietary nitrate can serve as an alternative source of NO (Lundberg et al., 2008). This “enterosalivary nitrate pathway” is driven by bacterial, rather than human, nitrate reductase enzymes residing within the oral cavity. Therefore the results of the current study may in part be explained by the antibiotic‐induced reduction of the commensal bacteria altering the circulating precursors available for NO.

To our knowledge, this is the first study to align colonic dysbiosis with cerebral endothelial dysfunction. The results of our study within the cerebral vasculature compliments previous work describing an important role of the gut microbiota in transmitting signals to the brain via the so‐called “gut‐brain axis” (Carabotti et al., 2015). It also aligns with previous work showing that manipulation of the gut microbiota affects both the structure and function of certain regions within the brain (Cowan et al., 2018). Our results in the cerebral vasculature parallel to the findings of Karbach et al. who documented a blunted endothelium‐dependent vasorelaxation in aorta harvested from colonized germ‐free mice compared to Swiss Webster mice (Karbach et al., 2016). In contrast, other studies in the peripheral vasculature describe an improvement in endothelium‐dependent vasorelaxation with a concomitant increase in eNOS protein expression after antibiotic treatment in mouse aorta (Vikram et al., 2016) and no effect in carotid artery (Brunt et al., 2019). Interestingly, these latter studies both used the same antibiotic cocktail of ampicillin, neomycin, metronidazole, and vancomycin.

### 4.4 Effect of antibiotic treatment on eNOS activity

We then sought to determine whether eNOS activity is blunted in pooled cerebral arteries harvested from antibiotic‐treated rats. To this end, immunoblotting was used to assess the ratio of phosphorylated eNOS to total eNOS as a surrogate marker for eNOS activity. Our results demonstrate that the eNOS‐P/total eNOS ratio was significantly reduced in antibiotic‐treated rats (Figure 6b). This data supports the premise that eNOS activity is reduced in cerebral arteries following antibiotic treatment.

The clinical ramifications of endothelial NO dysfunction have been well documented. Loss of endothelial NO can prime the cerebral vasculature for stroke (Feigin et al., 2002), aneurysms (Tamura et al., 2009), and Alzheimer's disease (Austin & Katusic, 2020). Therefore maintaining or improving optimal endothelial function is an important preventative approach to avoid many age‐related diseases. As the main triggers of endothelial dysfunction, quelling inflammation, and oxidative stress, can restore endothelial NO function (Siti et al., 2015). The results from the current study would suggest that our focus should shift to the influence of gut dysbiosis and inflammation in playing an integral role in mediating cerebral endothelial NO dysfunction. Interestingly, this communication is bi‐directional as evidenced in recent studies showing that stroke can in fact trigger gut dysbiosis (Xu et al., 2021) and that recolonization of the gut with healthy microbes leads to improved outcomes (Chen et al., 2019; Lee et al., 2020).

There were some limitations in the present study. First, the number of rats in each group was limited and therefore the results should be interpreted with caution. Second, alteration of the gut microbiota was performed with an antibiotic regimen that is, unlikely to be simulated clinically. While we know that gut dysbiosis is likely to affecting health in some patients, it is impossible to precisely replicate dysbiosis in an experimental model. Finally, further studies are needed to uncover the mechanisms linking gut dysbiosis with cerebral endothelial dysfunction. Examining the cerebral vascular effect of restoring the microbiome by replacing antibiotic water with normal water would also be very insightful.

The results of this study highlight the potential of the microbiota to communicate with the cerebral vasculature. Our findings suggest that dysbiotic perturbations can potentially elicit endothelial dysfunction thereby increasing risk of stroke and aneurysms. Taken together, this work underscores the importance of future medical treatment in adopting a whole body systems approach.

In conclusion, our data supports the role of the microbiota in driving cerebral endothelial dysfunction. Although our data are preliminary and warrants replication, it provides an exciting area of investigation. It highlights the potential of the microbiota as a target to reverse endothelial dysfunction and a preventative approach to reducing risk of stroke and aneurysms. The gut microbiota offers an opportunity that may be amenable to therapeutic approaches such as reducing burden (toxins, fungal overgrowth, parasites) and promoting beneficial growth (prebiotics, probiotics, and a low inflammatory diet).

## AUTHOR CONTRIBUTIONS

EMS designed the study. EMS and JSP designed the methodology. AJR and EMS performed the laboratory experiments. EMS, AJR, and GB analyzed the data. All authors contributed to writing the manuscript and approved the submitted version.

## Supporting information



Fig S1

Fig S2
