# Supplementary material for: Microbial disruption in the gut promotes cerebral endothelial dysfunction
Source: Physiol Rep. 2021 Nov 9;9(21):e15100. doi: 10.14814/phy2.15100 (PMC8578899; doi:10.14814/phy2.15100)
Supplement: Supplementary file 1 — Fig S1 [file PHY2-9-e15100-s002.pptx]

## Slide 1
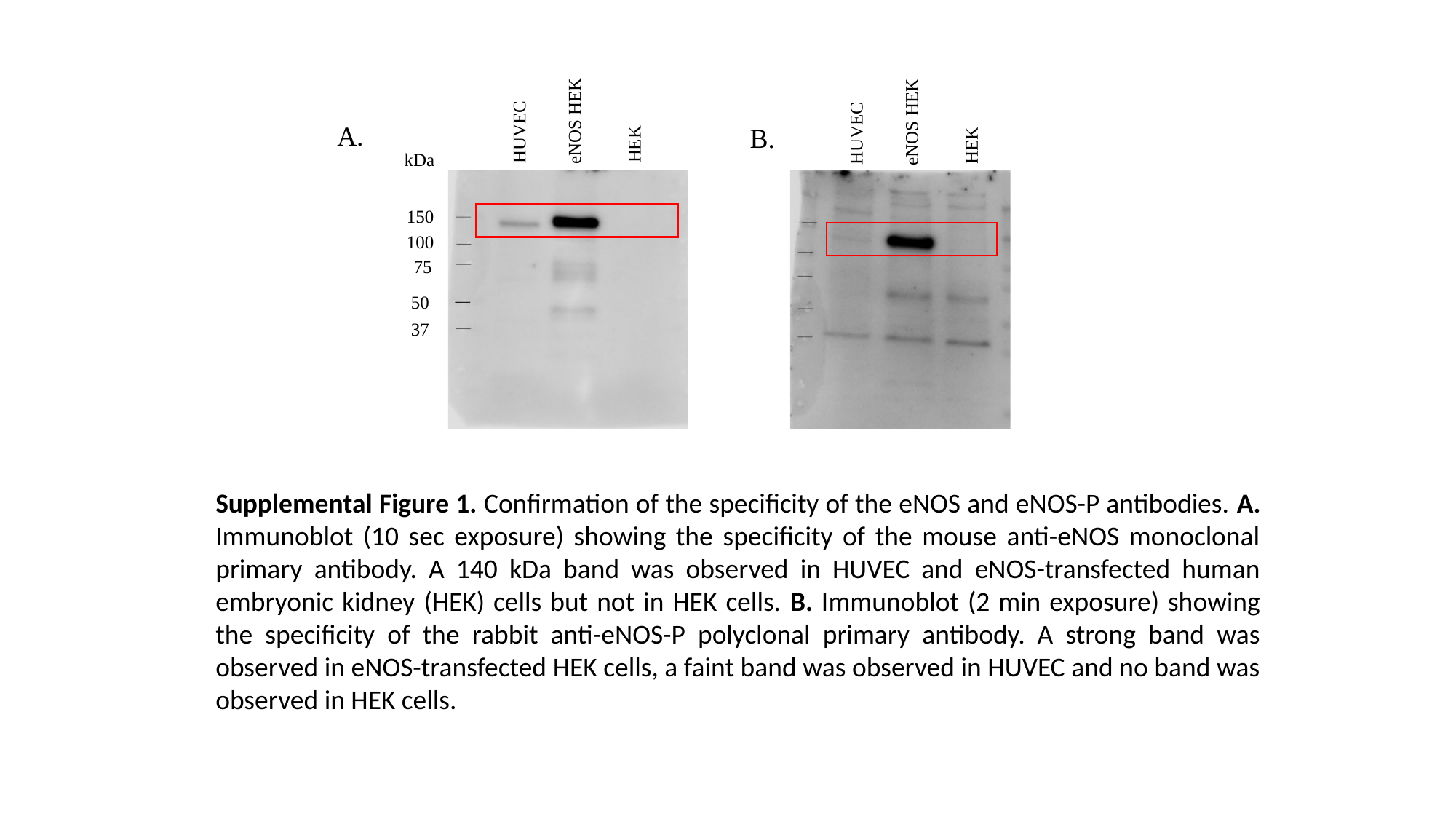

eNOS HEK
eNOS HEK
A.
B.
HUVEC
HUVEC
HEK
HEK
kDa
150
100
75
50
37
Supplemental Figure 1. Confirmation of the specificity of the eNOS and eNOS-P antibodies. A. Immunoblot (10 sec exposure) showing the specificity of the mouse anti-eNOS monoclonal primary antibody. A 140 kDa band was observed in HUVEC and eNOS-transfected human embryonic kidney (HEK) cells but not in HEK cells. B. Immunoblot (2 min exposure) showing the specificity of the rabbit anti-eNOS-P polyclonal primary antibody. A strong band was observed in eNOS-transfected HEK cells, a faint band was observed in HUVEC and no band was observed in HEK cells.
