# Supplementary material for: Microbial disruption in the gut promotes cerebral endothelial dysfunction
Source: Physiol Rep. 2021 Nov 9;9(21):e15100. doi: 10.14814/phy2.15100 (PMC8578899; doi:10.14814/phy2.15100)
Supplement: Supplementary file 2 — Fig S2 [file PHY2-9-e15100-s001.pptx]

## Slide 1
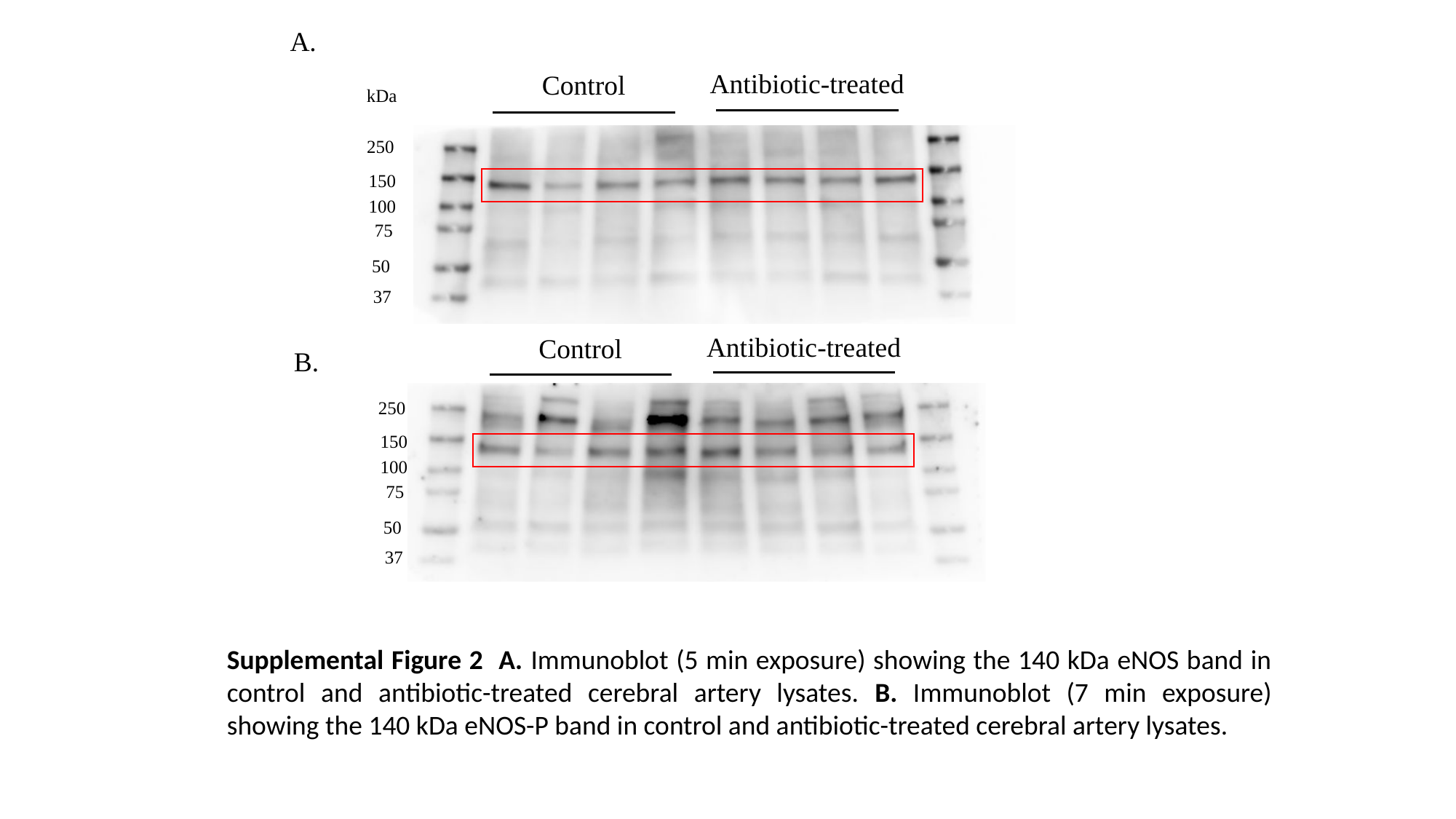

A.
Antibiotic-treated
Control
kDa
250
150
100
75
50
37
Antibiotic-treated
Control
B.
250
150
100
75
50
37
Supplemental Figure 2 A. Immunoblot (5 min exposure) showing the 140 kDa eNOS band in control and antibiotic-treated cerebral artery lysates. B. Immunoblot (7 min exposure) showing the 140 kDa eNOS-P band in control and antibiotic-treated cerebral artery lysates.
